# Development of a Mobile Health Snacktivity App to Promote Physical Activity in Inactive Adults (SnackApp): Intervention Mapping and User Testing Study

**DOI:** 10.2196/41114

**Published:** 2023-05-22

**Authors:** James P Sanders, Kajal Gokal, Jonah J C Thomas, Jonathan C Rawstorn, Lauren B Sherar, Ralph Maddison, Colin J Greaves, Dale Esliger, Amanda J Daley, Snacktivity Investigators

**Affiliations:** 1 Centre for Lifestyle Medicine and Behaviour Loughborough University Loughborough United Kingdom; 2 School of Sport, Exercise and Health Sciences Loughborough University Loughborough United Kingdom; 3 Institute of Physical Activity and Nutrition School of Exercise and Nutrition Science Deakin University Burwood Austria; 4 National Institute for Health Research Leicester Biomedical Research Centre Leicester United Kingdom; 5 School of Sport, Exercise and Rehabilitation Sciences University of Birmingham Birmingham United Kingdom; 6 See Acknowledgments

**Keywords:** intervention development, physical activity, mobile health, mHealth, health app, user testing, intervention, behavior, smartphone, app, social, user, engagement, development, testing, mobile phone

## Abstract

**Background:**

Despite the unequivocal evidence demonstrating the benefits of being physically active, many people do not meet the recommended guidelines of at least 150 minutes of moderate- to vigorous-intensity physical activity per week. This can be changed with the development and implementation of innovative interventions. The use of mobile health (mHealth) technologies has been suggested as a mechanism to offer people innovative health behavior change interventions.

**Objective:**

This study aims to outline the systematic, theory-driven processes and user testing applied to the development of a smartphone-based physical activity app (SnackApp) to promote participation in a novel physical activity intervention called Snacktivity. The acceptability of the app was explored and reported.

**Methods:**

Intervention mapping involves a 6-step process, the first 4 of which were presented in this study. These steps were used to develop the SnackApp for use within the Snacktivity intervention. The first step involved a needs assessment, which included composing an expert planning group, patient and public involvement group, and gathering the views of the public on Snacktivity and the public perception of the use of wearable technology to support Snacktivity. This first step aimed to determine the overall purpose of the Snacktivity intervention. Steps 2 to 4 involved determining the intervention objectives, the behavior change theory and techniques on which the intervention is based, and the development of the intervention resources (ie, SnackApp). After the completion of steps 1 to 3 of the intervention mapping process, the SnackApp was developed and linked to a commercial physical activity tracker (Fitbit Versa Lite) for the automated capture of physical activity. SnackApp includes provisions for goal setting, activity planning, and social support. Stage 4 involved users (inactive adults, N=15) testing the SnackApp for 28 days. App engagement (mobile app use analytics) was analyzed to determine app use and to inform the further development of SnackApp.

**Results:**

Over the study period (step 4), participants engaged with SnackApp an average of 77 (SD 80) times. On average, participants used the SnackApp for 12.6 (SD 47) minutes per week, with most of the time spent on the SnackApp dashboard and engaging, on average, 14 (SD 12.1) times, lasting 7 to 8 minutes per week. Overall, male participants used the SnackApp more than female participants did. The app rating score was 3.5 (SD 0.6) out of 5, suggesting that SnackApp was rated as fair to good.

**Conclusions:**

This study outlines and reports data regarding the development of an innovative mHealth app using a systematic, theory-driven framework. This approach can guide the development of future mHealth programs. User testing of the SnackApp suggested that physically inactive adults will engage with the SnackApp, indicating its applicability of use in the Snacktivity physical activity intervention.

## Introduction

### Background

The global population has become less physically active and more sedentary, and both behaviors are independently associated with poorer health outcomes and mortality [1-3]. The last two decades have seen a rapid increase in well-designed and robustly evaluated physical activity trials [4,5]. This has helped national governments and the World Health Organization to generate evidence-based physical activity recommendations [6,7]. In many countries, public health guidance recommends that adults should perform at least 150 to 300 minutes of moderate-intensity physical activity in bouts of any duration, as well as perform muscle-strengthening activities for at least 2 days a week [7]. However, few people achieve this amount of physical activity each week, and evidence suggests that initial increases in physical activity levels to meet guidelines are often not maintained over the long term [8-10]. Of particular concern are data from the United States and the United Kingdom that suggest that only 1% to 16% of adults participate in muscle-strengthening–based physical activities each week [11,12], which are important for reducing the risk of falls, fractures, and osteoporosis [13]. Another concern is the amount of time people spend being sedentary, with adults spending approximately 60% to 70% of their waking hours sitting [14]. For inactive adults, high levels of sedentary time have been associated with an increased risk of developing noncommunicable diseases (eg, type 2 diabetes) and all-cause mortality [15]. Together, this suggests that there is a need for more innovative interventions to encourage the population to regularly engage in physical activity and spend less time sedentary each day.

An alternative integrative approach to promoting physical activity that could engage and motivate people to be more physically active and break up sedentary time is a concept called *Snacktivity* [16]. Rather than emphasizing at least 150 minutes per week of physical activity, Snacktivity focuses on promoting short but frequent bouts of moderate- to vigorous-intensity physical activity (MVPA; known as *activity snacks*) throughout the day, such that people accumulate at least 150 MVPA minutes over the week. An *activity snack* lasts between 2 and 5 minutes, for example, walking during coffee breaks, squats while waiting for the kettle to boil, lunges while vacuuming, or getting off the bus a stop early or parking further away [16]. Other types of short-bout physical activity interventions already exist such as high-intensity interval training [17]; however, Snacktivity differs in purpose, duration, and intensity as an intervention approach. High-intensity interval training tends to be approximately 60 to 90 seconds in duration and at a physical intensity close to 100% exertion. In contrast, the Snacktivity approach aims to encourage the public to perform activity snacks in 2- to 5-minute bouts at moderate to vigorous intensity. Snacktivity is a multicomponent intervention approach that also encourages the public to incorporate or embed Snacktivity into everyday activities. Snacktivity is also designed to be an accessible approach to promoting physical activity in all populations, including individuals who are disabled and those who are living with chronic disease and or conditions, pregnant individuals, and older people.

### Mobile Health Interventions to Promote Physical Activity in the Population

To achieve a population-level increase in physical activity levels, we need effective interventions with high reach. Given their ubiquitous use, mobile health (mHealth) interventions can reach many people, and they are relatively inexpensive to deliver and can be offered at a convenient time and place [18]. Therefore, these tools can be used to promote physical activity interventions such as Snacktivity in the population [19]. Furthermore, mHealth interventions can integrate key health behavior change strategies, including self-monitoring, goal setting, prompts or nudges, and feedback on behavior, which are effective in increasing participation in physical activity [20]. Evidence from systematic reviews supports the use of mHealth interventions to promote health behavior changes [18,21,22], although trials with long-term follow-up (eg, >6 months) are still required [23,24].

### Intervention Mapping

Despite mHealth technologies becoming increasingly accessible, the development strategies and processes used to create interventions using them remain unclear. Appropriate steps are essential when developing evidence- and theory-based mHealth interventions to assist with effective implementation in real-world settings [25]. mHealth interventions must use a common language and a systematic and robust development process [20], including the use of theoretical frameworks (such as the intervention mapping [IM] framework) [26] to ensure that interventions are theoretically sound and can be reproduced and scaled up.

### Aims

This study aimed to outline the systematic, theory-driven processes (using IM) undertaken to develop a smartphone-based physical activity app (called SnackApp), which is linked to a wearable consumer physical activity tracker (Fitbit) to promote participation in Snacktivity. In addition, the acceptability of the app as measured by the level of user engagement with the SnackApp was explored and reported.

## Methods

### Overview

Following the UK Medical Research Council’s guidance for developing complex health care interventions [25,27], we used a systematic, evidence-informed approach to develop SnackApp. The development of SnackApp is part of a larger research program to test the effectiveness of Snacktivity in increasing physical activity across the day in adults [16]. In brief, the SnackApp (and accompanying consumer physical activity tracker) aims to support users to incorporate Snacktivity into their day through self-monitoring, by providing feedback on the number of activity snacks they have completed, by offering users a method to set goals for Snacktivity, and by creating action plans for completing activity snacks each day.

### SnackApp Development

**Figure 1:**
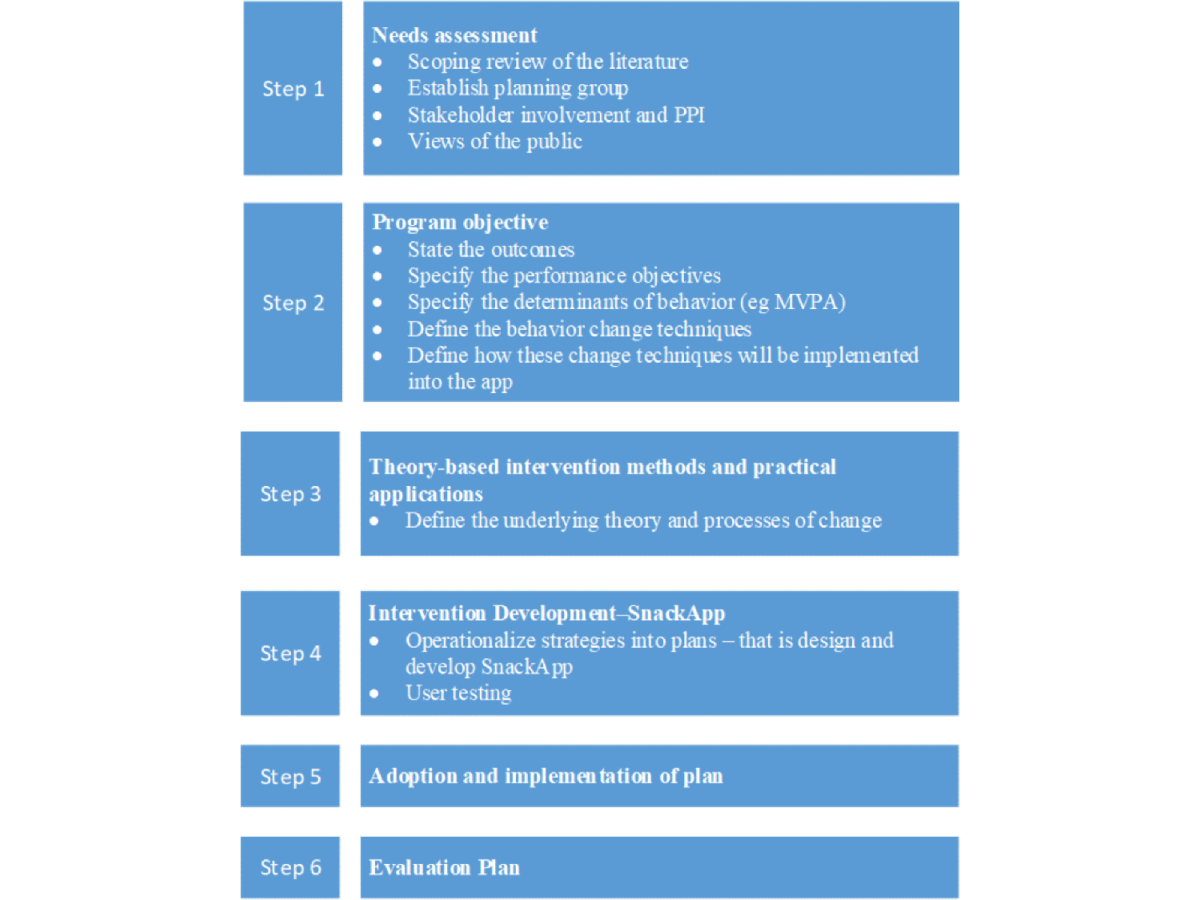
SnackApp development—intervention mapping overview. Steps 5 and 6 are ongoing [28]. MVPA: moderate- to vigorous- intensity physical activity; PPI: patient and public involvement.

IM Framework [26] is a 6-step framework, often applied to guide the development of health behavior change interventions. The first four stages of the IM development process (which are presented in this paper) include (1) conducting a needs assessment, (2) specifying intervention outcomes and objectives, (3) designing the intervention and applying theory, and (4) refining intervention development. The final 2 steps for SnackApp development (adoption, implementation, and evaluation plan) are currently being researched [28] (Figure 1).

### Step 1: Needs Assessment

#### Overview

As part of the initial steps to develop SnackApp, the research team reviewed relevant literature on behavior change theory and techniques related to physical activity promotion and mHealth interventions. This helped us guide the content (ie, key behavioral strategies and techniques, app implementation, and engagement) and focus of SnackApp. The development of the SnackApp coincided with the 2019-2020 update of UK physical activity guidelines, which removed the caveat suggesting that bouts of MVPA lasting ≥10 minutes were needed to derive health benefits (ie, acknowledging that any duration of MVPA is indeed health enhancing) [6].

Following this change in the physical activity guidelines and based on experimental evidence indicating that short bouts of MVPA may improve indicators of health [13,29-31], a consensus was reached from the planning group (see *Establish and Work with a Planning Group* section) that the SnackApp should aim to promote short bouts of MVPA (2-5 min), in line with the behavioral principles of Snacktivity, such that users accumulate their weekly total minutes of MVPA through the accumulation of activity snacks.

#### Establish and Work With a Planning Group

A multidisciplinary team involving 16 individual psychologists, experts in physical activity and digital health, behavioral scientists, medical sociologists, and health care professionals was established to provide their expertise in the development and content of the SnackApp and to agree on the physical activity behaviors to be measured and the information to be feedback to users. A total of 33 meetings between some or all members of the planning group were held between September 2019 and December 2020. During these meetings, the expert planning group undertook a series of tasks described below in step 3 (eg, MoSCoW [Must Have, Should Have, Could Have, and Won’t Have] framework) to determine the content, design, and behavior change techniques (BCTs) within the SnackApp.

#### Stakeholder and Patient and Public Involvement

During the early development of the Snacktivity intervention, 165 members of the public responded to a questionnaire regarding the concept of Snacktivity and the use of digital technology as a mechanism for behavior change. Furthermore, 3 focus groups (n=10; 6/10, 60% female) were conducted to explore the concept of Snacktivity. The focus group attendees discussed the importance of embracing digital technologies to enable people to lead healthier lifestyles. One theme that was generated related to the concept that it is necessary to ensure that there is sufficient opportunity for members of the public to be a part of the development of the SnackApp and test it before it is used. Focus group participants described the app that they were using to increase their physical activity, and they wanted to ensure that the SnackApp incorporated the best features. These included motivational messages to encourage people to engage in more activity snacks and graphics showing their achievements (unpublished data). Furthermore, throughout the planning process, we worked closely with a group of public contributors (public advisory group [PAG]; N=11) who provided feedback on the development of SnackApp and the wider Snacktivity intervention [32].

#### View From the Public

Following the initial stakeholder and PAG feedback stage, a survey to further assess the views of the public about Snacktivity was completed by 754 members of the public in the West Midlands [33]. When the participants were asked what would help them to perform Snacktivity throughout the day, the most frequently selected options included seeing how much Snacktivity had been completed each day, having enough examples of activity snacks to do, and receiving an alert reminding them to do Snacktivity. Furthermore, when asked about physical activity trackers and mHealth app use, most participants (678/754, 89.9%) owned a smartphone, and of them, 45% (305/678) used their mobile phones to track their physical activity. Of this 45% population, 52.1% (159/305) downloaded an app to the phone to aid in tracking their physical activity, and the most appealing features were considered to be the option to track steps and the ability to view a summary of their data. The most commonly owned physical activity tracker was the Fitbit. The 3 most appealing features of an activity tracker were notifications to move when sedentary for too long, the ability to track physical activity, and the ease of use. On the basis of these findings, the expert group agreed that the intervention should aim to increase MVPA and include both an mHealth app and the Fitbit physical activity tracker for the Snacktivityintervention.

### Step 2: Specification of Outcomes, Performance Objectives, and Change Techniques

The second IM step involved developing the specific health BCTs and aligning the Snacktivity behaviors to BCTs in inactive adults. Informed by the findings from stage 1 (needs assessment), the planning group identified 3 key performance objectives of SnackApp. This included the objectives to be achieved by individuals so that they complete Snacktivity and MVPA (eg, engagement with the SnackApp) and the behavioral determinants and techniques that would influence their physical activity behavior (Table 1).

The overall purpose of SnackApp is to facilitate an increase in users’ total minutes of participation in MVPA each week by encouraging engagement with Snacktivity. The three performance objectives (the behavioral outcomes intended to be achieved that would promote participation in Snacktivity and the achievement of goals) for the SnackApp or consumer physical activity tracker were as follows:

1. Education on the principles of Snacktivity and why and how it could be important for health
2. Facilitation and sustainable engagement with the SnackApp
3. Promotion of participation in minutes of MVPA per day per week

Drawing from the literature and the behavior change taxonomy [34], we aimed to achieve these 3 performance objectives through the implementation of several BCTs within the SnackApp and Fitbit physical activity tracker [34]. The BCTs were incorporated into the SnackApp and aimed to increase modifiable determinants of MVPA (eg, self-efficacy) to achieve performance objectives. The BCTs included self-monitoring, goal setting, prompts or cues, and feedback (Table 1). With the performance objectives and outcomes decided, it was important to determine how these outcomes could be changed. The next step was to determine the BCTs that the literature had suggested as effective in improving physical activity behavior.

**Table 1:** Program outcomes, performance objectives, and behavior change techniques (BCTs) used in the development of the SnackApp.

Performance objective, modifiable determinants, and BCT				Snacktivity intervention outcome—increase in weekly minutes of MVPA^a^ (how BCTs were implemented into the app)
**Learn about Snacktivity and why and how it is important for health**				
	**Knowledge**			
		Provide information on the behavior	Written information on the health benefits of Snacktivity is provided in the form of articles on the SnackApp	
		Provide information on the consequences	Text on benefits of physical activity for health as a resource within the SnackApp	
		Salience of consequences	Emphasize the detrimental health consequences of physical inactivity via push notifications and written content in the app	
**Engagement with the SnackApp**				
	**Intention**			
		SnackApp personalization^b^	Users engage with SnackApp functions tailored to their needs. Automatic and tailored goal setting based on their previous MVPA behavior Personalized the metrics in which the users are interested. A choice between the number of activity snacks, active minutes, and steps	
		Credible source	Users will know the SnackApp has been created by health behavior change experts	
**Increase daily MVPA**				
	**Self-efficacy**			
		Prompt specific goal setting^c^	The user is prompted to use the goal-setting function in the SnackApp to formulate their own goals related to steps, active minutes, and activity snacks	
		Graded task^c^	Users can use automatic goal-setting functionality within the app, which will automatically increase or decrease user goals based on previous data as a substitute for manual goal setting	
		Prompt barrier identification^d^	Users can create action plans within the app about how to complete activity snacks throughout the day	
		Prompt review of behavioral goals^c^	Regular review of progress and increase goal for duration and bouts of physical activity by the user	
		Behavior instruction or demonstration	Exposure to modeled behavior. The SnackApp has examples of activity snacks, with videos and instructions on how they should be performed	
		Individualized feedback on MVPA and steps and activity snacks^c^	Numerical feedback (graph and push numbers) on the SnackApp will provide data on users’ Snacktivity behavior	
		Focus on past success^c^	Historical graphical data displaying instances when users successfully increased their physical activity	
		Self-monitoring of behavior^c^	Encourage monitoring of own behavior, encourage users to check daily whether they have reached their Snacktivity goals via push notifications	
		Prompts and cues^c,d^	Prompts or push notifications for the individual to break up prolonged periods of inactivity or sedentary behavior as measured by the activity tracker	
		Habit formation^d^	Provision for users to anchor planned activity snacks to existing behaviors (eg, when I brush my teeth, I will do squats for 2 minutes)	
		Social reward^c^	Congratulatory push notification on reaching a goal	
		Reframing^d^	Framing motivational messages such as “some is good more is better”	
		Relapse prevention^d^	Provide motivational messages to promote MVPA after a period of relapse	
	**Subjective norms**			
		Social support^c,d^	Provision of a forum within the app for individuals to discuss their Snacktivity	
		Social comparison	Optional provision for users to see how their peers are performing in comparison with themselves	
	**Enjoyment**			
		Offer users an option of performing Activity Snacks they enjoy^b^	Users to choose from a library of activity snacks that they enjoy	

^a^MVPA: moderate- to vigorous-intensity physical activity.

^b^These are the BCTs, which were determined by the expert planning group based on their expertise and the Patient Advisory Group from their experiences.

^c^These BCTs are related to control theory and self-regulation theory.

^d^These BCTs are derived from habit formation model.

### Step 3: Theory-Based Intervention Methods and Practical Applications

#### Overview

Step 3 of the IM process involved the selection of BCTs targeting each modifiable determinant identified in Table 1. For each determinant (ie, knowledge, self-efficacy, and enjoyment) of each performance objective (eg, increasing daily MVPA per minute), BCTs that were likely to alter the determinant (eg, self-monitoring) were selected [26]. This stage was based on the existing BCT taxonomy [34]. To ensure that different perspectives were included in this development stage, the PAG was asked, through a series of workshops, to comment on the strategies that they had found to be successful in encouraging them to be more physically active and were asked to review the BCTs that were planned to be included in the SnackApp. This increased the likelihood that they would be feasible and acceptable for potential users. In addition, as part of the process of determining which BCTs should be included, we used a MoSCoW method exercise with the PAG and the expert planning group. Briefly, MoSCoW stands for “Must have,” “Should have,” “Could have,” and “Won’t have,” and is a prioritization technique used in software development to reach a common understanding with stakeholders on the importance they place on the BCT components and strategies to be included. Members of the expert planning group were provided with a series of BCTs and how they could be implemented into the SnackApp and were asked to complete the SnackApp MoSCoW template. Responses that were deemed “Must” or “Should haves” BCTs were incorporated into the SnackApp.

#### Underlying Theory or Process of Change

The SnackApp is based on several theoretical perspectives. The key principles in the development of SnackApp were to encourage user learning from their experience through engagement in self-regulation activities (eg, self-monitoring, feedback, and goal setting), in line with control theory [35] and self-regulation theory [36]; build intrinsic motivation; and promote autonomy (also consistent with self-determination theory) [37]. An important aim of SnackApp is to promote the formation of daily habits for Snacktivity in line with the habit formation model [38] to maximize the potential for the maintenance of Snacktivity and physical activity. With the BCTs decided, the next step focused on the inclusion and implementation of the BCTs within Snacktivity intervention resources, in this case, the SnackApp.

### Step 4: Intervention Development—SnackApp

#### Overview

Step 4 of the IM process was to develop the SnackApp. This step involved translating the BCTs (and the underlying processes of change) into specific SnackApp functionalities (eg, how to measure Snacktivity, how to provide feedback and goal setting, and what information about Snacktivity should be included in the SnackApp). The planning group discussed and guided the scope, selection of behavioral strategies (ie, how BCTs would be translated into app features), and the feasibility of encouraging Snacktivity via an app. To facilitate the initiation and adoption of Snacktivity, the SnackApp was developed to integrate a range of BCTs that used different app elements (ie, push notifications, data, graphs, and a planner for activity snacks; Table 1).

#### Snacktivity and Physical Activity Tracking

For users to self-monitor their goals and receive feedback about their Snacktivity, decisions needed to be made about how physical activity should be measured and how users’ progress from the SnackApp was presented and fed back to facilitate their engagement with Snacktivity. One way to achieve this is by using device-based measures of physical activity (eg, accelerometers, fitness trackers, and smartwatches) that can record physical activity continuously. Among the many different consumer wearable physical activity monitors (based on the findings from step 1), the Fitbit Versa smartwatch (Fitbit Inc) was selected. Fitbit Versa has onboard computing that allows data to be analyzed instantaneously to ensure immediate access, feedback, and rewards to users regarding the completion of their Snacktivity goals to promote and facilitate continued engagement with the SnackApp.

#### SnackApp: Mobile App Development

The research team, working with an app development company, created a series of designs on how the BCTs would be incorporated into SnackApp. These designs were based on modern design techniques (user experience) and previously reviewed apps from commercial and research spaces. These designs were piloted among the planning groups and the participant advisory group and were discussed until a consensus was reached on the best options available.

**Figure 2:**
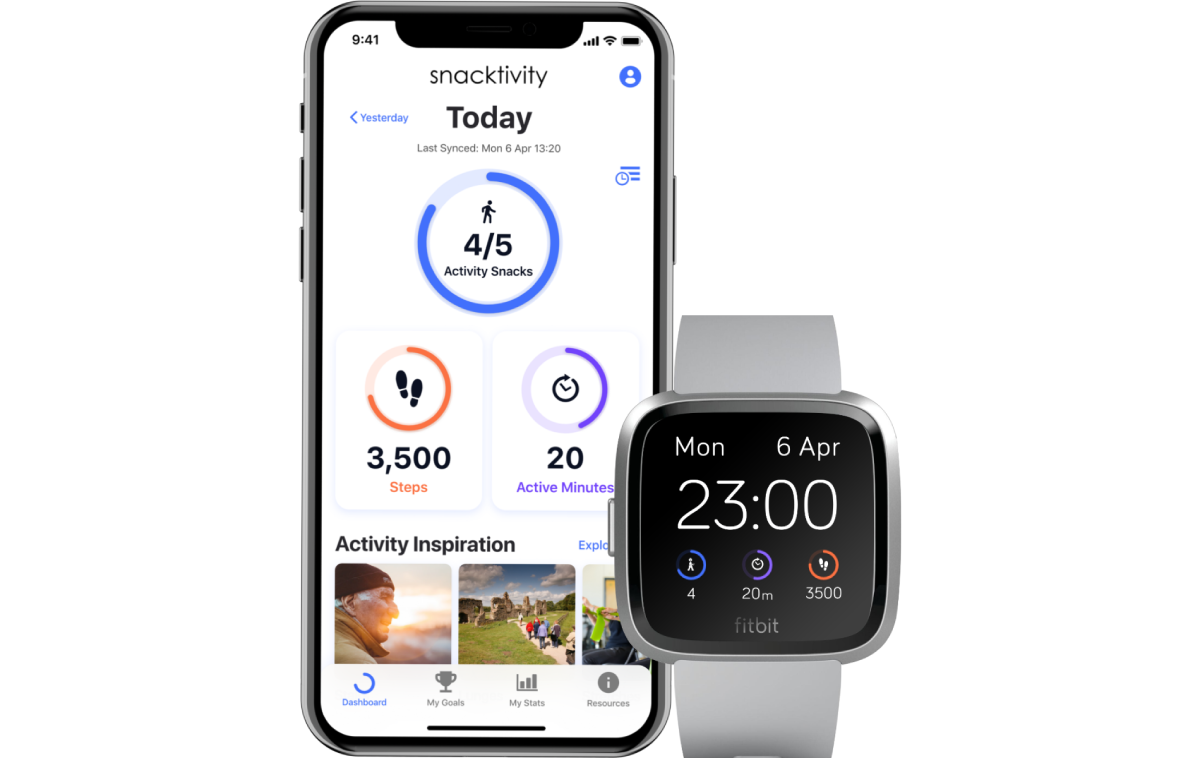
SnackApp and Fitbit clockface.

The SnackApp (Figure 2) is divided into five main areas as follows:

1. “Dashboard”—the main screen of the SnackApp where users can self-monitor their daily activity metrics and progression toward their daily goal
2. “My Stats”—A historical look at the user’s activity to provide feedback on their metrics across days, weeks, and months, also with progression toward their goals
3. “My Goals”—users can set their activity goals relating to activity snacks, steps, and active minutes. Within this area, users can also set action plans related to their goals. Action plans are structures to guide and encourage users to identify when, what, where, and how long the desired behavior change will be conducted (eg, when I am brushing my teeth, I will perform squats for 2 minutes).
4. “My Resources”—this area contains useful examples of activity snacks and how they can be performed safely, articles on the importance of Snacktivity and being physically active, and frequently asked questions regarding the use of the SnackApp and the activity monitor. A “forum” section was included in this area where users of the SnackApp can send messages to each other to discuss Snacktivity and provide social support to engage in Snacktivity.
5. “Notifications”—these formed 2 types: SnackApp and clockface notifications. These notifications were used to provide prompts, cues, or nudges to encourage users to be active.
  - App push notifications: push notifications were grouped into 3 main types: relapse prevention, motivational, and informational; they were prioritized to be delivered in that order. Relapse prevention notifications were designed to guide people in using the app. Motivational messages were designed to help people reach their goals, congratulate participants when they have reached their goals, or encourage them if they have failed to meet their daily targets. Informational messages would be used to tell users that the battery on their watch was running low to ensure that they were charged and therefore in use.
  - Clockface notification: in addition to the push notifications sent to the phone, nudges or prompts could also be displayed on the clockface. After a period of inactivity, the watch would display a message to encourage users to perform an activity snack. If the user was to perform an activity snack within 2 minutes of this prompt, a congratulatory message would appear (Multimedia Appendix 1 provides further information).

There was an aspect of customization to these prompts, as participants could personalize the duration of time before an inactivity prompt was provided (maximum 90 min), as well as the time frame in the day when the SnackApp would send prompts (eg, between 9 AM and 5 PM). These customizations meant that the users were only being nudged in the way decided by them to guard against frustration and disinterest with the SnackApp.

### Calculation of Activity Snacks

Activity snacks are defined as short bouts of MVPA lasting 2 to 5 minutes. Fitbit uses the Karvonen formula to determine activity intensity and returns this as active minutes within their system. Therefore, 2 to 5 active minutes would be considered an activity snack.

### Ethics Approval and Informed Consent

This study received favorable ethical opinion from the London City and East Research Ethics Committee (20/PR/0323). Eligible participants were sent a participant information sheet, and they provided written informed consent before any data collection took place.

### User Testing of the SnackApp

The next stage of the IM process was to obtain user feedback on SnackApp to allow further development and refinement.

#### Design, Setting, and Participants

Physically inactive adults who had completed a previous work package within the Snacktivity program [33] were recruited to test the first version of SnackApp. Participants were defined as inactive based on their responses to the General Practice physical activity questionnaire. Those who did not use a commercial physical activity tracker but did own an Apple or Android smartphone compatible with iOS 10+ or Android 4.1+ were recruited.

#### Data Collection

Once participants had provided informed consent, they were sent the Fitbit Versa Lite via post. Participants were also sent instructions on how to download, install, sign in, and connect to the SnackApp and Fitbit app and were asked to incorporate Snacktivity into their daily lives for 28 consecutive days while wearing the Fitbit (with the Snacktivity watch face) and using the SnackApp. All data were anonymized before analysis.

#### Measures

##### User Version of the Mobile Application Rating Scale

Participants were asked to complete the user version of the mobile application rating scale (uMARS [39]) and report their answers specifically concerning their experiences of using the SnackApp at the end of the data collection period. There are 3 components assessed by the uMARS: “App Quality” (engagement, functionality, esthetics, and information); “App Subjective Quality” (whether participants would recommend the app to others); and perceived impact (whether the app has increased the participant’s awareness, knowledge, attitudes, intention to change, help seeking, and behavior change).

##### User Engagement

Engagement with consumer physical activity trackers and mHealth apps is not well defined in the literature [40], and there is no standardized list of metrics that can be described and measured for engagement with mHealth apps. Therefore, we defined user engagement as the participants’ use and interactions, captured directly via the SnackApp. Furthermore, the “Display on/off” time (ie, the amount of time the Fitbits screen was on or off henceforth called “Clockface queries”) was measured to provide a proxy indication of the time spent interacting at the SnackApp clockface.

#### Data Analyses

User engagement was collated and analyzed to provide data on the number of bouts (per day) and the duration of time that users accessed the SnackApp, as well as the time spent on each of the specific screens within the SnackApp. Furthermore, the number and duration of time users viewed the SnackApp clockface as displayed on the Fitbit were summarized, and any bout duration of “clockface display on” >5 seconds was used as a proxy for engagement with the SnackApp information displayed on the Fitbit; 5 seconds was selected for pragmatic reasons because it was considered the minimum time when people could be reasonably assumed to be looking at the SnackApp clockface and to distinguish from the time people might be only determining the time of day, which typically takes only 1 to 2 seconds.

A Sankey analysis diagram was generated using the SnackApp user engagement data. Sankey diagrams (the outcome of a Sankey analysis) are visualizations used to depict the flow from one set of values to another. Sankey analysis are best used in mobile analytics to show “traffic” flows from one app page to another. Each link between the “node” represents a change in the screen viewed in the SnackApp, whereas each “node” represents a particular screen within the SnackApp.

Data relating to user engagement with SnackApp and SnackApp tracker watch clockface on the Fitbit were downloaded from bespoke customer relationship management software and analyzed using proprietary software (The SnackApp Usage package) developed in R Statistical Computing Language (R Foundation for Statistical Computing), which was further developed into a Shiny application [41,42]. The source code for the app (SnackApp Usage package version 0.9.2) can be found on GitHub [43,44].

The subscale and total scores for the uMARS were calculated. The total quality score of the SnackApp was determined by averaging the mean scores of the engagement, functionality, esthetics, and information subscales. The app’s subjective quality subscale items were reported individually. Given the purpose of this research, it was not appropriate to conduct statistical means difference or relationship testing; instead, summary data are presented.

## Results

### Participants

A total of 15 adults (female, n=8, 53%; male, n=7, 47%; mean age: mean 53, SD 15.7 years; mean self-reported BMI 27.3, SD 4.2 kg/m^2^) took part in the study (Table 2).

**Table 2:** Participant demographics (N=15).

		Values
Age (years), mean (SD)		53.0 (15.7)
BMI (kg/m^2^), mean (SD)		27.3 (4.2)
**Sex, n (%)**		
	Male	7 (47)
	Female	8 (53)
**Self-reported ethnicity, n (%)**		
	White	10 (67)
	Black	2 (13)
	Pakistani	3 (20)

### Engagement

#### SnackApp

**Figure 3:**
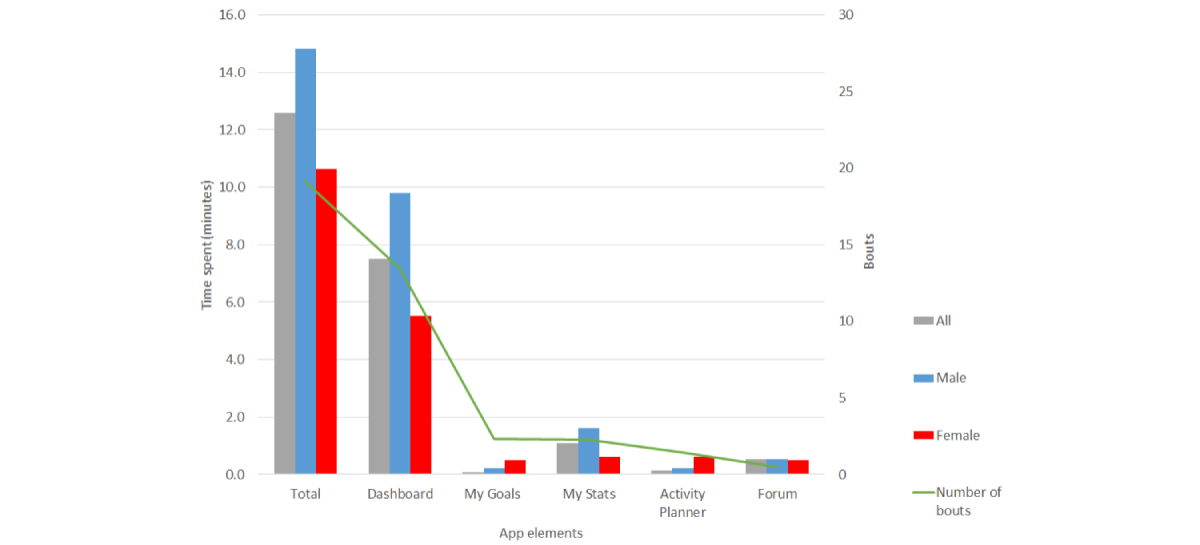
Average time spent (minutes) and number of instances on the SnackApp and individual elements of the SnackApp per week.

Over the 28-day study period, participants engaged with the SnackApp on average 77 (SD 80) times, for an average total SnackApp time use of 12.6 (SD 47) minutes per week, with most of this time spent on the SnackApp dashboard (14, SD 12.1 bouts lasting 7.5, SD 31 min/week). On average, engagement with the SnackApp was higher among male individuals than among female when considering the overall use (male=24 instances, 15 min/week vs female=15 instances, 11 min/week) and all individual app elements, except for “my goals” (male=2 instances, 1.5 min/week; female=3 instances, 0.6 min/week) and “activity planner” (male=0.2 instances, 0.6 min; female=2 instances, 0.6 min/week; Figure 3).

**Figure 4:**
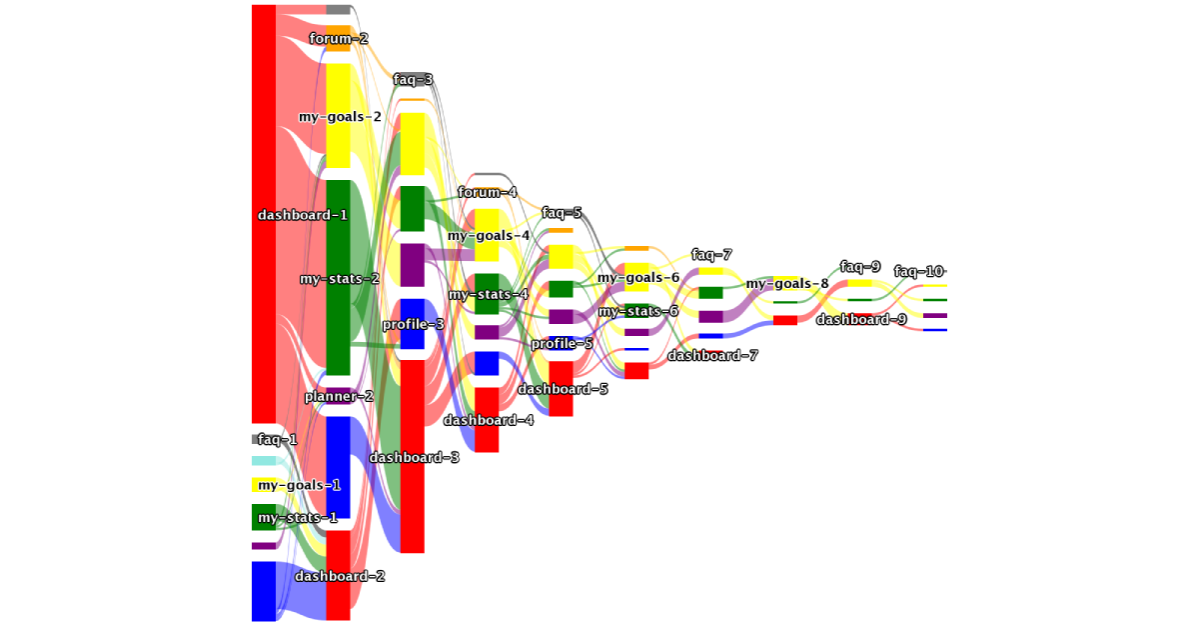
Sankey diagram representing the flow of participants through the SnackApp from the first page on opening the app through to the 10th page subsequently. Red: dashboard; gray: FAQ; yellow: my goals; green: my stats; purple: activity planner; blue: profile; and amber: forum. FAQ: frequently asked questions. Code available on Github.

Figure 4 shows a Sankey diagram, which illustrates that most SnackApp interactions began on the dashboard (default landing page) and typically progressed to the “my stats” and “my goals” pages. Users, who did not initially interact via the dashboard, typically engaged with the dashboard as the second page. The “activity planner” and “forum” pages tended to be the third or fourth pages engaged with on the SnackApp. The height of the column on the Sankey diagram (Figure 3) indicates the number of users who interacted with the app in that order. For example, column 5 is shorter than column one, as fewer users made ≥5 interactions with the SnackApp. The Sankey diagram shows that there are a diminishing number of users interacting with the app after the fifth column, suggesting that most users performed 5 interactions with the app before exiting the app.

#### Physical Activity Monitor (Fitbit)

Participant SnackApp clockface was queried 112 times per day, of which 52 queries were sustained for >5 seconds (accumulating a median time of 8.4 min spent querying the clockface per day). Almost 80% of the total time querying the SnackApp clockface display was accounted for in the sustained queries (Table 3).

**Table 3:** Fitbit and SnackApp clockface queries.

			Values, median (range)
**Daily clockface queries (frequency per day)**			111.7 (34-285)
	Male	97.4 (34-152)	
	Female	153.9 (60-285)	
**Sustained^a^ clockface queries (number per day)**			51.8 (23-105)
	Male	40.0 (34-152)	
	Female	64.7 (60-285)	
**Duration (sustained minutes^a^ or total min/day) **			8.4 (3.6-28.9)
	Male	8.2 (3.8-9.2)	
	Female	11.1 (3.6-28.9)	

^a^Sustained queries are defined as the clockface being on for 5 seconds or longer.

#### uMARS Score

The mean overall app quality score was 3.5 (SD 0.6) out of 5, with the highest mean component score for esthetics and information (mean score 3.6). Results from the perceived impact subscale showed that participants “agreed” that the SnackApp increased their awareness, knowledge, and attitude toward increasing physical activity as well as their intention to change and behavior change (Table 4).

**Table 4:** User version of the mobile application rating scale results from the SnackApp.

		Mean score (SD; out of 5)	Score definition
**App quality score**		3.5 (0.6)	—^a^
	Engagement	3.4 (0.7)	—
	Functionality	3.3 (0.7)	—
	Esthetics	3.6 (0.6	—
	Information	3.6 (1.0)	—
**App subjective quality**			
	Would you recommend this app to people who might benefit from it?	3 (1.4)	Maybe
	How many times would you use the app in the next 12 months use?	4 (1.2)	10-50
	Would you pay for this app?	1 (0.5)	Maybe
	What is your overall star rating of the app?	3.5 (0.5)	Average
**Perceived impact**			
	Awareness	4.2 (1.4)	Agree
	Knowledge	3.8 (1.4)	Agree
	Attitudes	4.1 (1.4)	Agree
	Intention to change	4.0 (1.2)	Agree
	Behavior change	4.2 (0.9)	Agree

^a^Not available.

## Discussion

### IM Framework

This report presented a systematic method that combines conceptual and technological frameworks in the development of a new health app called SnackApp, which will contribute to the future development of the Snacktivity intervention. Specifically, we have described the practical application of the IM framework to the development of Snacktivity intervention by identifying the behaviors of interest (ie, main behavioral facilitators of physical activity and sedentary behavior) and the application of specific BCTs to bring about health behavior change using mHealth technologies.

### User Testing

User testing of the SnackApp focused on utility and engagement, intending to increase users’ enjoyment and motivation of the app [45,46]. Our approach provided an understanding of how participants used and moved through the SnackApp, such that the intended outcome of the behavior change intervention (eg, completing activity snacks) was optimized.

The number of times users need to engage with mHealth interventions for effective behavior change to occur, across different populations, contexts, and time frames, is unknown. Our data indicated that users engaged with the individual elements of the SnackApp to varying degrees, with the dashboard, stats pages, and goal-setting elements being those that participants interacted with the most. This is encouraging, given that these components of the app encompass the BCTs of goal setting, feedback on progression toward goals, and self-monitoring, which previous research has shown as key ingredients in facilitating physical activity behavior changes [20]. The average total SnackApp time was 12.6 minutes per week. It is currently unknown what level of engagement with digital health intervention would be needed to initiate and sustain behavior change, but participants would likely need to engage with the SnackApp more, in the short term, to sustain any behavior change and to form habits. That said, Yardley et al [47] argued that paradoxically, as habits are formed, this may result in lower use of the SnackApp and users become less reliant on the intervention for behavior change.

Ratings of subjective app quality indicated that participants considered the SnackApp to be of fair to good quality. Although the average engagement score of uMARS was 3.5 out of 5, users indicated that they did not find SnackApp entertaining. The “entertainment” aspect of the “app engagement” score was 3 out of 5 (which suggests that the app was “OK, fun enough to entertain user for a brief time [<5 minutes]”). While this is only a modest rating of the app’s entertainment, entertainment is not the main function of SnackApp. Implementing or increasing enjoyment when using health apps is difficult because enjoyment is not their primary role. However, other health apps have included gamification opportunities, often through avatars, unlocking of areas or levels within the app after a certain period or as a reward for progression and competition among users. It may be that efforts should be made as part of future iterations of SnackApp to include such features.

### Strengths and Limitations

This study describes in detail the key perspectives of IM that informed the development of SnackApp through the combination of evidence; participation and engagement from patients, public, and academic stakeholders; and the use of behavioral change theories and BCTs. Overall, our development work was based on a well-established and comprehensive theoretical framework. It is also important to highlight that although the needs assessment and evidence synthesis used here were based predominantly on findings from systematic reviews conducted previously, our underpinning evidence of mHealth use for health behavior change was also based on rigorous studies that had evaluated digital programs.

Several technological strengths relating to the SnackApp merit further discussion. The SnackApp has been designed using the React Native platform (Meta Platforms Inc). This platform enables the production of 1 app across multiple mobile platforms (eg, iOS and Android), meaning the app can reach a larger population. In addition, it is cheaper to develop (and subsequently maintain and update) 1 code base rather than 2 separate code bases for each platform. Furthermore, the use of the Fitbit Versa smartwatch series provides the potential for data to be collected, analyzed, and fed back to participants, increasing the capability for immediate feedback on Snacktivity behavior to users. This is important because Snacktivity is an approach that is likely to be most potent when participants engage with it throughout the whole day, so the need for instantaneous feedback is beneficial. In addition, the SnackApp system collected comprehensive data about the engagement of users within the SnackApp and with the Fitbit clockface. In the future, these data will be important for understanding how users engage with Snacktivity to understand any mediators to participation in Snacktivity ^TM^.

This study has some limitations. The study analysis focused on how and whether users would engage with the SnackApp, but as participants were only followed for 28 days, it is difficult to determine the level of attrition over a longer period (eg, 1 year). Indeed, within the mobile app industry, many engagement and attrition metrics are calculated over several months of use rather than 1 month. Future studies should assess the feasibility and effectiveness of such interventions. Furthermore, SnackApp currently connects only to Fitbit Versa series devices. This will affect the ability of SnackApp to scale up the population level. Future iterations of SnackApp should integrate it across a wider range of wearable devices.

### Future Research and Implications

The IM framework is a useful resource that helped to develop a robust digital self-monitoring program for our target audience. Future studies should consider assessing the feasibility and effectiveness of SnackApp and accompanying SnackApp watch face over a longer period.

Although the behavioral goal setting was individually and dynamically tailored to each participant’s physical activity behavior patterns, tailoring may be enhanced by considering additional variables, such as age, sex, health literacy, self-efficacy, intention, and environmental factors (eg, weather patterns), which are likely to affect physical activity behavior and may lead to higher personal relevance of the SnackApp to participants and subsequently contribute to long-term behavior change [48-50]. Furthermore, our user testing was on a small population with a vested interest in Snacktivity; therefore, it would be useful to assess the usability of SnackApp in a larger, more diverse population. As part of future research, a qualitative study could seek to understand the experiences (thoughts and feelings) participants had while using the SnackApp, which would support its further development.

The results of user testing and feedback will be used to inform modifications to the SnackApp. For example, feedback suggests that there is a need to increase the enjoyment of the app to help maintain engagement. This can be achieved through the gamification of the app. Furthermore, clear messaging could be provided in the app on how to complete an activity snack in terms of the intensity and duration of activities. Furthermore, SnackApp currently uses Fitbit to measure participants’ Snacktivity. Future research should look to specifically validate Fitbit as a measure of short bouts of physical activity.

### Conclusions

The IM framework was used to aid the development of a theory- and evidence-based mHealth intervention called Snacktivity. IM contributed to the identification of the determinants and optimal theoretical methods to promote participation in Snacktivity. User testing was also conducted, suggesting that physically inactive adults will engage with the SnackApp. Future research needs to be conducted to assess how engagement with SnackApp will change over time, as well as contribute to intervention effectiveness.

## Appendix

Multimedia Appendix 1 SnackApp technical developments: additional information.

## Abbreviations

BCT: behavior change technique
IM: intervention mapping
mHealth: mobile health
MoSCoW: Must Have, Should Have, Could Have, and Won’t Have
MVPA: moderate- to vigorous-intensity physical activity
PAG: public advisory group
uMARS: user version of the mobile application rating scale

## Data and Code Availability

The data sets generated and analyzed during this study are available from the corresponding author on reasonable request. Software application or custom code is available on the internet [44].
