# Supplementary material for: Development of a Mobile Health Snacktivity App to Promote Physical Activity in Inactive Adults (SnackApp): Intervention Mapping and User Testing Study
Source: JMIR Form Res. 2023 May 22;7:e41114. doi: 10.2196/41114 (PMC10242461; doi:10.2196/41114)
Supplement: Multimedia Appendix 1 [file formative_v7i1e41114_app1.docx]

Supplementary File 1 – SnackApp Development

There are three apps to consider here. The native Fitbit app, a companion app (explained below) and the newly developed SnackApp. The content of the SnackApp was designed to be adaptive, can be tailored to an individual’s preferences, and responsive to their achievement of activity snacks. The SnackApp was developed (via a commercial app development company) using React Native (Facebook Inc, California, USA), and is written in JavaScript language. SnackApp was initially developed over four months. The accompanying Fitbit Versa physical activity tracker collects various measures of physical activity data and displays these data using a bespoke SnackApp clockface (see Figure 1) via the Fitbit Software Development Kit (SDK) written in JavaScript. The SnackApp clockface on the tracker watch, which is set as default, provides immediate feedback on the number of activity snacks, ‘active minutes’ (i.e., MVPA) and the number of steps users have achieved. The clockface on the Fitbit tracker watch connects to the Fitbit app via a companion app, which is used to enhance any native Fitbit app by providing it with an additional JavaScript runtime environment that exists within the Fitbit mobile application. This companion app can utilise resources and sensors of the mobile phone which extends the data processing capability of the Fitbit app. With an internet connection, the companion app uploads the data to the Snacktivity™ application programming interface (API) where they are stored in PostgreSQL databases hosted on a secure encrypted Google server. The SnackApp then reads the following information from the database upon opening or refreshing the app:

1. individual information (i.e., profile), such as active minutes, steps, and activity snacks, for feedback and to help calculate the individualised activity profile and daily goals for the user.
2. a list of intervention elements to be delivered to the user upon certain conditional rules (e.g., push notification alerting users that they have completed a goal); and
3. a library of intervention content/elements (i.e., intervention repository) such as activity snack examples, images, articles and forum messages to be displayed.

The SnackApp is divided into five main areas:

1. ‘Dashboard’ – the main screen of the SnackApp where users can self-monitor their daily activity metrics and progression towards their daily goal
2. ‘My Stats’ – A historical look at the user's activity to provide feedback on their metrics across days, weeks, and months, also with progression towards their goals
3. ‘My Goals’ – Users can set their activity goals relating to activity snacks, steps, and active minutes. Within this area, users can also set action plans relating to their goals. Action plans are structures to guide and encourage users to identify when, what, where and how long the desired behaviour change will be conducted (e.g., when I am brushing my teeth, I will perform squats for two minutes)
4. ‘My Resources’ – This area contains useful examples of activity snacks and how they can be performed safely, articles on the importance of Snacktivity™ and being physically active, and Frequently Asked Questions (FAQs) regarding the use of the SnackApp and the activity monitor. Also, the Resources section has a ‘forum’ section in this area, users of the SnackApp can send messages to each other within the platform to discuss Snacktivity™ and provide social support to get active and engage in Snacktivity™
5. Notifications – these formed two groups: SnackApp and clockface notifications. These notifications were used to provide prompts/cues/nudges to users to encourage them to be active.
   1. App push notifications: Push notifications were grouped into three main types; relapse prevention, motivational and informational and were prioritised to be delivered in that order. Relapse prevention notifications were designed to guide people back to using the app. For example, if a user has not opened the app in two days, a push notification will be sent to the phone with the message *‘Success is easier if you track your Snacktivity™ {Participant First Name}! Check-in with your SnackApp today.’* Clicking on the message would direct them to the SnackApp. Motivational messages were designed to help people reach their goals, congratulate participants when they have reached their goals or encourage them if they have failed to meet their daily targets. Examples include *‘Fantastic [Participant Name} That’s your Activity Snack goal complete! Keep up the good work!’* or if they have not met their goal *‘Every minute counts! Here are some great activity snacks option which you can do anywhere!’* Informational messages would be used to tell users that the battery on their watch was running low, in an attempt to make sure they keep charged and therefore in use.
   2. Clockface notification: In addition to the push notifications sent to the phone, nudges/prompts could also be displayed on the clock face. These prompts were designed to encourage participants to perform an activity snack. After a period of inactivity, the watch would display a message (e.g. *Inactivity alert! Snacktivity™ time!’*) to encourage users to perform an activity snack. If the user was to perform an activity snack within two minutes of this prompt, a congratulatory message would appear (e.g. *‘Well done on your activity snack!*).

There was an aspect of customisation to these prompts, as the participants could personalise the duration of time before an inactivity prompt was provided (max 90 minutes), as well as the window of time in the day in which the SnackApp could send prompts (e.g. between 9am and 5pm). These customisations meant the users were only being nudged in a way that they decided, which was believed to guard against participants becoming frustrated or disenfranchised with the SnackApp.

### Calculation of Activity Snacks.

Activity snacks are defined as short bouts of MVPA lasting 2-5 minutes. Fitbit utilises the Karvonen formula to determine activity intensity and return this as active minutes within their system. Therefore 2-5 active minutes would be considered an activity snack.
